# UV laser mediated cell selective destruction by confocal microscopy

**DOI:** 10.1186/1749-8104-3-11

**Published:** 2008-04-28

**Authors:** Laurent Soustelle, Benoît Aigouy, Marie-Laure Asensio, Angela Giangrande

**Affiliations:** 1 Institut de Génétique et Biologie Moléculaire et Cellulaire, IGBMC/CNRS/ULP/INSERM – BP 10142 67404 ILLKIRCH, c.u. de Strasbourg, France

## Abstract

Analysis of cell-cell interactions, cell function and cell lineages greatly benefits selective destruction techniques, which, at present, rely on dedicated, high energy, pulsed lasers and are limited to cells that are detectable by conventional microscopy. We present here a high resolution/sensitivity technique based on confocal microscopy and relying on commonly used UV lasers. Coupling this technique with time-lapse enables the destruction and following of any cell(s) in any pattern(s) in living animals as well as in cell culture systems.

## Background

Cell selective destruction provides a fruitful approach to study cell-cell interactions, cell function and cell lineages [1,2]. Laser assisted techniques provide fine control over the selective destruction time during development and allow for targeting of any cell(s) in any pattern(s), which is hardly feasible using genetic approaches [3]. This technique, based on the use of pulsed lasers that destroy cells by high energy deposition, has allowed lineage mapping in *Caenorhabditis elegans *and has been extensively applied to the large cells of the grasshopper nervous system [1,4,5]. This technique was also used in *Drosophila *to study different processes, such as embryonic morphogenetic movements [6,7] or the development of the nervous [8] and muscular systems [9]. Until recently, such a technique, however, has been limited to cells that can be identified using Nomarski optics. Although this limit has been partially overcome by the advent of green fluorescent protein (GFP) based transgenesis and the development of epifluorescence set-ups, low levels of GFP expression in the targeted cells and/or high levels of autofluorescence still restrict the use of laser assisted cell selective destruction. We here describe a technique that combines UV laser mediated cell destruction and confocal technology. This technique, which relies on the use of common lasers, allows for high resolution and sensitivity and works efficiently in cell cultures as well as in living animals.

## Results and discussion

### UV-mediated destruction of *Drosophila *cells

Currently used laser assisted techniques for selective cell destruction are based on pulsed lasers and conventional microscopes. To circumvent the limits of such an approach (low signal level, autofluorescence), we have devised a simple and efficient UV laser and confocal based technique and used it to study glial development in *Drosophila *pupal wings. Upon production of a wing glial precursor, proliferation and migration account for the formation of a continuous sheath along axonal fibers, a process that is necessary for proper transduction of the electric signal to the central nervous system (for a review, see [10]). In order to analyze the cell-cell interactions controlling both migration and proliferation, it was essential to destroy specific glial cells and monitor the behavior of the remaining ones. Using the UAS-GAL4 system [11], we generated transgenic animals labeling glial cells *in vivo*. Flies carrying *repo-gal4 *[12], a glial specific driver, and the *UAS-ncGFP *reporter, which allows for GFP expression in the nucleus and the cytoplasm, were collected at pupal stages at which wing glia move and divide. *repo-gal4, UAS-ncGFP *(*repo::GFP*) pupae were taped and the puparium case laying over the wing removed using scissors. The exposed tissue was covered with 10S halocarbon oil (Voltalef^®^, Prolabo, Fontenay s/bois, France) to prevent it from drying. The animal was subsequently transferred onto a Glass-Bottom dish (Willco-dish™, Amsterdam, Netherlands) and analyzed using a Leica TCS SP2 inverted confocal microscope (Leica, Heidelberg, Germany) equipped with an Ar laser (488 nm) to excite GFP and a UV laser (351 nm and 364 nm) to perform the selective destruction. A heating stage (Heating Insert P, Pecon, controller Tempcontrol 37-2 digital, Pecon [13]) was used to maintain a constant temperature (25°C ± 2°C). In our study, using confocal microscopy is crucial for identifying the cells to target, as optical sections remove parasite signals coming from different focal planes and allow for the detection of low intensity GFP signals.

The protocol used for cell selective destruction by confocal microscopy is as follows. The cell to be ablated is identified based on GFP expression and scanned in the Z axis to locate the center of the nucleus. Within the selected focal plane, using the 'Point bleach' function of the LEICA software targets a region of the nucleus. This option, which is generally also used to perform FRAP experiments, is available on non-Leica confocal microscopes and can thus be generally used. The 'Point bleach' function permits the selection and targeting of a specific area along the XY axes within a single Z focal plane. The area can vary from a single pixel to a larger surface that can be chosen depending on the application. In addition, several individual pixels can be simultaneously targeted, which permits the use of different irradiation times for each selected position. In our case, we selected a single pixel in the 512 × 512 pixel image observed under the confocal microscope and submitted it to 20 seconds of UV laser excitation (351 nm and 364 nm).

UV laser intensity was measured using a NovaII powermeter (Ophir [14,15]), upon placing the detector head in a special 'slide-shaped' device. Measurements were made after calibration of the whole system, including the confocal microscope, UV laser and powermeter. We used the same objective as that used for cell destruction (Leica 63X HCX Plan Apo CS, NA 1,4, lambda blue) and placed the objective at its upper position (Additional file 1). At maximum power, we obtained the following values: 186 μW at 351 nm and 168 μW at 364 nm (maximum values). We also took measurements using the back aperture of the objective, which is more reproducible and universal (because it is objective independent) and detected a power value of 2.3 mW. This is an important point to note, as we have observed that, at lower power intensities, UV-induced cell destruction is not fully penetrant (data not shown). For optimal excitation, the UV adaptation optics (UV lens button) must be set prior to each selective destruction. UV lens selection is used to fill the back aperture of the objective for maximum control of focusing in the Z axis. The consequences of UV irradiation are monitored by time-lapse on the whole animal.

**Figure 1:**
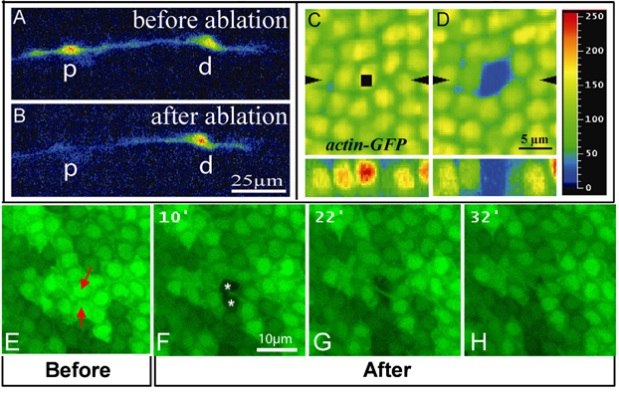
High resolution images of confocal assisted UV laser selective destruction. **(a,b) **Confocal images of *repo::GFP *expressing cells prior to (a) and after (b) selective destruction. (b) The proximal glial cell (p) has been targeted for selective destruction; the distal cell (d) was not targeted. **(c,d) ***actin-GFP *labeling in the fly wing epithelium prior to (c) and just after (d) UV irradiation. The region of interest defined by the 'Point bleach' function is indicated by the black square in (c). (d) Notice that, upon irradiation, GFP labeling is specifically absent in the targeted nucleus. Images to the bottom of (c,d) correspond to Z optical sections taken along the axis indicated by the black arrowheads. Color coding is used to quantify GFP labeling: blue (0) corresponds to background, red (250) to high levels. **(e-h) ***actin-GFP *labeling in the fly wing epithelium prior to (e) and 10 (f), 22 (g) or 32 minutes (h) after UV irradiation. The two targeted cells (indicated by red arrows in (e)) are absent 10 minutes after UV-mediated destruction (indicated by white asterisks in (f)). The space previously occupied by the targeted cells is subsequently occupied by the neighboring cells (g,h). See also Additional file 5. Scale bars: (a,b) 25 μm; (c,d) 5 μm; (e-h) 10 μm.

After 20 seconds of UV irradiation (power at maximum values, speed 400 Hz), GFP labeling rapidly fades (within minutes) and never resumes, even after several hours (Figure 1a,b), suggesting that the targeted cell has died. Death induced by 20 seconds of UV irradiation has been confirmed by lack of glial-specific labeling in the targeted cell (Additional file 2; see also [16]). Altogether, these data strongly suggest that UV laser targeting leads to cell destruction. To formally conclude that cell death occurs only in the irradiated cell, we have performed several control experiments.

**Figure 2:**
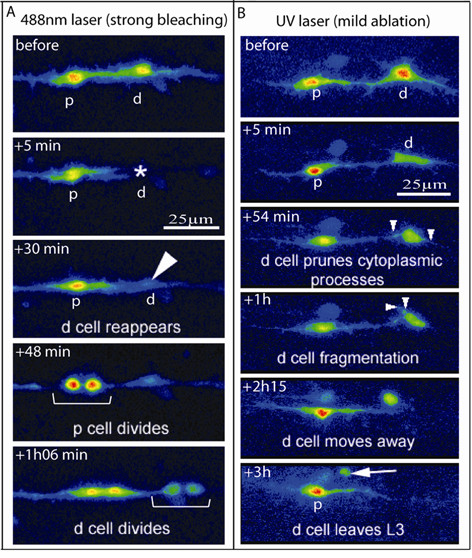
Effects of UV irradiation versus bleaching. **(a,b) **Color coding of confocal images of *repo::GFP *expressing cells in strong bleaching (a) or mild UV irradiation (b) experiments. (a) The distal glial cell (d) has been targeted using the 488 nm ray. GFP completely disappears from the cell within 5 minutes after bleaching (asterisk) and subsequently resumes (arrowhead). Notice that both targeted (d) and non-targeted (p) cells divide and that they do it almost simultaneously. See also Additional file 3. (b) The distal cell (d) has been targeted for mild UV irradiation; 54 minutes to 1 hour afterwards, the d cell prunes its cytoplasmic processes and undergoes fragmentation (small arrowheads). Finally, between 2.25 and 3 hours after UV irradiation, the d cell loses contact with its neighbors, acquires a round shape and moves away (arrow). See also Additional file 4. Scale bars: 25 μm.

First, to exclude the possibility that death is induced by bleaching, we performed experiments using a 488 nm beam. In this case, even when the strongest bleaching conditions were used, GFP labeling disappeared but rapidly resumed (Figure 2a; Additional file 3). These bleaching conditions did not produce any apparent damage as targeted cells divide and migrate the same as non-targeted cells, therefore behaving as wild-type cells (Figure 2a; Additional file 3).

Second, to further confirm the different cellular behaviors induced by the two wavelengths, we used mild UV irradiation conditions (10 seconds), which allow the targeted cell to retain some GFP expression and to be followed over time (Figure 2b). After 10 seconds of UV irradiation, the targeted cell undergoes rounding, fragmentation and loses contact with neighboring cells (Figure 2b; Additional file 4).

Third, using transgenic animals that express GFP ubiquitously (*actin-GFP*) has allowed us to show that only the targeted cell lacks GFP labeling, and is destroyed by 20 seconds of UV irradiation (Figure 1c,d). The space occupied by the targeted cells is eventually occupied by its neighbors (Figure 1e–h; Additional file 5).

**Figure 3:**
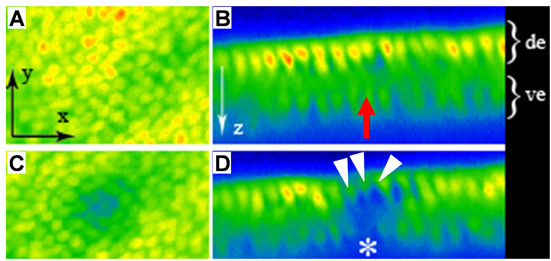
UV cell destruction of ventral wing epithelial cells does not affect dorsal cells. **(a-d) **GFP labeling prior to (a,b) and after (c,d) UV cell destruction in fly wing epithelium. Dorsal views are shown in (a,c). The wing epithelium presents two cell layers as shown on transversal sections in (b,d). Dorsal epithelium (de) is to the top, ventral epithelium (ve) to the bottom. In each panel, acquisition is made from dorsal to ventral. The same color coding as described in Figure 1 is used to quantify GFP labeling. Before (b) and after (d) irradiation, the ventrally located targeted cell is indicated by a red arrow (b) and a white asterisk (d), respectively. Note that the dorsal GFP-positive cells (white arrowheads in (d)) located just above the ventral targeted cell are still present upon UV irradiation, even though they display a reduced fluorescence signal. See also Additional file 6.

Finally, to show that UV irradiation does not trigger death at layers located above the targeted cells, we irradiated ventrally located cells in the fly wing epithelium of *actin-GFP *animals (red arrow in Figure 3b) and followed dorsally located cells (white arrowheads in Figure 3d) by time-lapse (Additional file 6). GFP-positive cells located on the dorsal epithelial sheet were not affected by UV-mediated cell destruction of ventrally located cells, as shown in Figure 3b,d) and Additional file 6, even though they show weak bleaching (Figure 3b,d).

### UV-mediated destruction of HeLa cells

**Figure 4:**
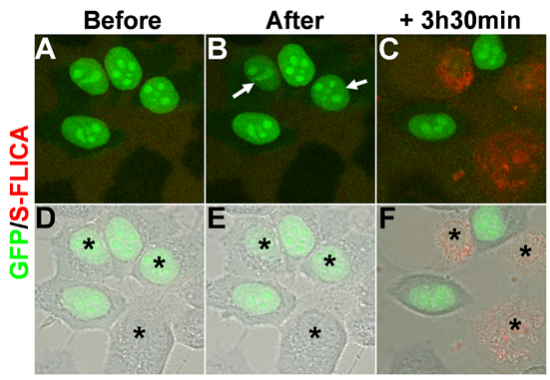
Confocal assisted cell destruction of HeLa cells. **(a-f) **Confocal images of HeLa cells transfected with pCIG nuclear GFP-expressing vector prior to (a,d), and just after (b,e) cell destruction, and after 3.5 hours of time-lapse analysis (c,f). Before UV irradiation, the S-FLICA apoptosis marker was added to the medium, becoming red in the presence of active caspases. (d-f) Merged images of GFP (green)/S-FLICA (red) colabeling with Nomarski images. Black asterisks indicate the two GFP-positive and the GFP-negative cells that have been targeted for ablation. Note that after 5 seconds of UV irradiation within the nucleus, weak GFP bleaching occurs in targeted GFP-positive cells (white arrows in (b)). After 3.5 hours, all the targeted cells (but not non-targeted cells) express active caspases, as indicated by red labeling. See also Additional file 7.

In order to show the general applicability of the confocal UV laser selective destruction method, we established a mammalian cell culture system by transfecting HeLa cells with a nuclear GFP-expressing vector. Cell culture analyses have also allowed us to further explore the selective destruction conditions and mechanisms inducing death. Cells were submitted to UV irradiation 48 hours after transfection. We reasoned that monolayer cells are more accessible than fly wing epithelium and, therefore, reduced the length of the UV pulse to 5 seconds; the other parameters remained the same as those mentioned above. The center of the nucleus was targeted using the 'Point bleach' function and the 63X oil objective, and the behavior of targeted cells was subsequently followed by time-lapse over several hours. In all cases (n = 20 in all experimental conditions), we observed that GFP bleaches and totally disappears with time (Figure 4; Additional file 7), again indicating that cell death has occurred. Moreover, to determine the cause of cell death, we took advantage of a cell permeable and non-cytotoxic red fluorochrome inhibitor that reveals apoptotic cells (called sulforhodamine-FLICA (S-FLICA)) [17]. Recent studies have shown that the S-FLICA signal is due to its binding to active caspases and to other unknown proteins associated with apoptosis [18]. The use of this substrate allowed us to establish that laser-induced death occurs via apoptosis, which may be mediated by caspase activation. A red signal appears 30–190 minutes after UV irradiation and increases with time (Additional file 7). Importantly, the labeling kinetics were similar in GFP-negative and GFP-positive cells (Figure 4; Additional file 7), indicating that GFP expression has no influence on the UV-induced cell death. This was confirmed by the observation that GFP signal intensity does not influence the death process since high and low GFP-expressing cells displayed the same apoptotic kinetics upon UV irradiation (data not shown). Finally, the S-FLICA signal is specific to treated cells as cells that were not UV irradiated did not show any labeling, even 3.5 hours after irradiation (Figure 4). S-FLICA labeling was observed in all targeted cells (n = 20). Altogether, these data show that HeLa cells UV irradiated for 5 seconds die by apoptosis. Similar results were obtained after 10, 15 or 25 seconds of UV irradiation; in these cases, death markers appeared faster than after 5 seconds of irradiation. Finally, 1 second UV irradiation did not seem to trigger cell death, since targeted cells did not display any morphological phenotype nor labeling for active caspases 4.5 hours after UV irradiation (data not shown).

## Conclusion

We describe a technique allowing for fine control over the destruction of cells not detectable by conventional microscopy. We provide quantitative parameters for targeting cells, and we show the fate of the targeted cells and highlight the molecular pathway induced by the UV irradiation. While current approaches are based on high energy deposition, which can be provided by pulsed lasers but not by the lasers normally equipping confocal microscopes, our technique is based on a widely used laser and exploits the damaging capacity of UV. Finally, the fact that cells of different types and sources can be successfully targeted indicates that this technique has a wide applicability in the field of biology.

## Materials and methods

### Fly strains

Flies were raised at 25°C according to standard procedures. Transgenic animals of genotypes *repo-gal4 *[12] and *UAS-ncGFP *(nc: nuclear and cytoplasmic) [19] were crossed in order to specifically induce GFP expression in glial cells. Flies with ubiquitous *actin-GFP *driven expression were obtained from the Bloomington Fly Stock Centre [20].

### Animal preparation for time-lapse and image processing

Preparation of animals for time-lapse was done as described in [16]. Cells were imaged in four dimensions using a TCS SP2 inverted confocal microscope (Leica) equipped with a Ar laser (488 nm) to excite GFP and a UV laser (Coherent Enterprise ENTC651: 351 nm and 364 nm) to perform selective destruction. Z stack projections, color coding, rotations, figure mounting and two color time-lapse movies were obtained using in-house developed TIMT imaging software (details available upon request). In order to reduce noise, a median filter 3 × 3 was applied on red image stacks prior to projection. Finally, images were annotated using Adobe Photoshop and Adobe Illustrator, and movies were converted to the QuickTime format using Adobe Premiere.

### Laser power measurement at the back of the objective

The objective that was used for the laser ablation experiments (Leica 63X HCX Plan Apo CS, NA 1,4, lambda blue objective) was selected to set all the parameters (scanner parameters, UV lens). The objective was taken out by unscrewing it and the detector was placed on a specific holder on the stage (or on a glass slide). Without the objective, all the rays are parallel, thus avoiding a focus problem on the detector. The display of the NovaII powermeter was set to 351 nm and the point bleach option in the Leica confocal software was used to avoid acousto optic tunable filter (AOTF) blanking. The measurement was done after the point bleach was set in the center of the field of view/image.

### HeLa cell experiments

HeLa cells were plated in 35 mm glass base dishes and grown at 37°C in DMEM supplemented with 10% fetal bovine serum and antibiotics. HeLa cells were transfected with pCIG nuclear GFP-expressing vector by using Effecten Transfection Reagent (Qiagen, Courtabeuf, France). After 48 hours, medium was washed out to eliminate floating cells and 2 ml of fresh medium supplemented with 10 ml of 30X S-FLICA (Apoptosis Detection Kit Caspase Assay, Immunochemistry Technologies, Bloomington, Minneapolis, USA) was added. Then, transfected cells were treated with UV laser irradiation using a 63X objective. Analysis was performed by time-lapse confocal microscopy using a heating stage to maintain a constant temperature (37°C). Experiments were performed in three independent transfection assays.

## Abbreviations

GFP: Green fluorescent protein; S-FLICA: Sulforhodamine-FLICA.

## Competing interests

The authors declare that they have no competing interests.

## Authors' contributions

BA carried out the *in vivo *studies, LS and MLA performed HeLa cell experiments, and AG conceived the study, participated in its design and coordination and helped to draft the manuscript. All authors read and approved the final manuscript.

## Supplementary Material

Additional file 1 Laser power measurement. **(a-e) **To measure the laser power, we placed the detector head of a power meter (a) in a special 'slide-shaped' device (b), as shown in (c). The special head detector-containing slide-shaped device is then placed on the confocal stage (d) to measure the laser power with a NovaII power meter (Ophir) (arrow in (e)). With this setup, the head detector is illuminated like a classic slide for UV cell destruction experiments. To determine a reproducible position for the measurement, we placed the Leica 63X HCX Plan Apo CS, NA 1.4, lambda blue objective at its upper position. Power measurements after complete recalibration of the whole system (confocal microscope, UV laser and power meter) are 186 μW at 351 nm and 168 μW at 364 nm. A more reproducible and universal measurement (objective independent, see Materials and methods) at the back aperture of the objective gives a value of 2.3 mW. Click here for file

Additional file 2 Confocal assisted UV laser selective cell destruction. **(a,b) **Confocal images of *repo::GFP *expressing cells prior to (a) and after (b) selective destruction of glial cells in a developing *Drosophila *wing (indicated by the dotted area in (a,b)). Distal is to the right. (a) Cells targeted for selective destruction are indicated by arrows. (b) After UV irradiation (17 hours after puparium formation), GFP labeling rapidly faded, suggesting that targeted cells die (indicated by asterisks). (c) Repo immunolabeling on the same dissected wing at 22 hours after puparium formation. Cell death is confirmed by lack of Repo glial-specific labeling in the targeted cells (indicated by asterisks) whereas other glial cells (included in the dotted area) were not affected. Click here for file

Additional file 3 Wing glia GFP bleaching. Time-lapse sequence showing that strong bleaching conditions (using a 488 nm beam) do not produce any apparent damage as targeted cells divide and migrate just as non-targeted cells, therefore behaving as wild-type cells. Click here for file

Additional file 4 Wing glia mild UV irradiation. Time-lapse sequence showing that by using mild UV irradiation, the targeted cell, which retains some GFP expression, undergoes rounding, fragmentation and loses contact with neighbor cells. Click here for file

Additional file 5 Epithelial rearrangement after cell destruction. Time-lapse sequence showing that only the targeted cell lacks GFP labeling, and is destroyed upon UV irradiation. Note that the space occupied by the targeted cells is eventually occupied by its neighbors. Click here for file

Additional file 6 Selective destruction along the Z axis. Time-lapse sequence showing that GFP-positive cells located on the dorsal epithelial sheet are not affected by UV-mediated cell destruction of ventrally located cells. Click here for file

Additional file 7 Confocal assisted cell destruction of HeLa cells. Time-lapse sequence showing that UV-mediated destruction of HeLa cells induces apoptosis, as confirmed by S-FLICA labeling. Click here for file
