# Supplementary figures and images for: UV laser mediated cell selective destruction by confocal microscopy
Source: Neural Dev. 2008 Apr 28;3:11. doi: 10.1186/1749-8104-3-11 (PMC2387153; doi:10.1186/1749-8104-3-11)

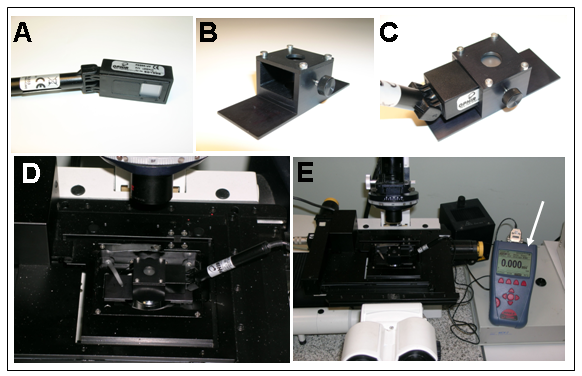

Supplement: Additional file 1 — Laser power measurement. (a-e) To measure the laser power, we placed the detector head of a power meter (a) in a special 'slide-shaped' device (b), as shown in (c). The special head detector-containing slide-shaped device is then placed on the confocal stage (d) to measure the laser power with a NovaII power meter (Ophir) (arrow in (e)). With this setup, the head detector is illuminated like a classic slide for UV cell destruction experiments. To determine a reproducible position for the measurement, we placed the Leica 63X HCX Plan Apo CS, NA 1.4, lambda blue objective at its upper position. Power measurements after complete recalibration of the whole system (confocal microscope, UV laser and power meter) are 186 μW at 351 nm and 168 μW at 364 nm. A more reproducible and universal measurement (objective independent, see Materials and methods) at the back aperture of the objective gives a value of 2.3 mW. [file 1749-8104-3-11-S1.tiff]

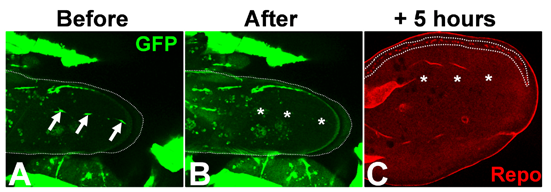

Supplement: Additional file 2 — Confocal assisted UV laser selective cell destruction. (a,b) Confocal images of repo::GFP expressing cells prior to (a) and after (b) selective destruction of glial cells in a developing Drosophila wing (indicated by the dotted area in (a,b)). Distal is to the right. (a) Cells targeted for selective destruction are indicated by arrows. (b) After UV irradiation (17 hours after puparium formation), GFP labeling rapidly faded, suggesting that targeted cells die (indicated by asterisks). (c) Repo immunolabeling on the same dissected wing at 22 hours after puparium formation. Cell death is confirmed by lack of Repo glial-specific labeling in the targeted cells (indicated by asterisks) whereas other glial cells (included in the dotted area) were not affected. [file 1749-8104-3-11-S2.tiff]
